# CTen: a web-based platform for identifying enriched cell types from heterogeneous microarray data

**DOI:** 10.1186/1471-2164-13-460

**Published:** 2012-09-06

**Authors:** Jason E Shoemaker, Tiago JS Lopes, Samik Ghosh, Yukiko Matsuoka, Yoshihiro Kawaoka, Hiroaki Kitano

**Affiliations:** 1 JST ERATO KAWAOKA Infection-induced Host Responses Project, Tokyo, Japan; 2 The Systems Biology Institute, Tokyo, Japan; 3 Influenza Research Institute, Department of Pathobiological Sciences, School of Veterinary Medicine, University of Wisconsin-Madison, Madison, Wisconsin, USA; 4 Institute of Medical Science, Division of Virology, Department of Microbiology and Immunology, University of Tokyo, Tokyo, Japan; 5 Sony Computer Science Laboratories, Inc, Tokyo, Japan; 6 Open Biology Unit, Okinawa Institute of Science and Technology, Okinawa, Japan

**Keywords:** Cell type enrichment, Microarray data, Deconvolution, Influenza, Systems immunology

## Abstract

**Background:**

Interpreting *in vivo* sampled microarray data is often complicated by changes in the cell population demographics. To put gene expression into its proper biological context, it is necessary to distinguish differential gene transcription from artificial gene expression induced by changes in the cellular demographics.

**Results:**

CTen (cell type enrichment) is a web-based analytical tool which uses our highly expressed, cell specific (HECS) gene database to identify enriched cell types in heterogeneous microarray data. The web interface is designed for differential expression and gene clustering studies, and the enrichment results are presented as heatmaps or downloadable text files.

**Conclusions:**

In this work, we use an independent, cell-specific gene expression data set to assess CTen's performance in accurately identifying the appropriate cell type and provide insight into the suggested level of enrichment to optimally minimize the number of false discoveries. We show that CTen, when applied to microarray data developed from infected lung tissue, can correctly identify the cell signatures of key lymphocytes in a highly heterogeneous environment and compare its performance to another popular bioinformatics tool. Furthermore, we discuss the strong implications cell type enrichment has in the design of effective microarray workflow strategies and show that, by combining CTen with gene expression clustering, we may be able to determine the relative changes in the number of key cell types.

CTen is available at http://www.influenza-x.org/~jshoemaker/cten/

## Background

**Figure 1:**
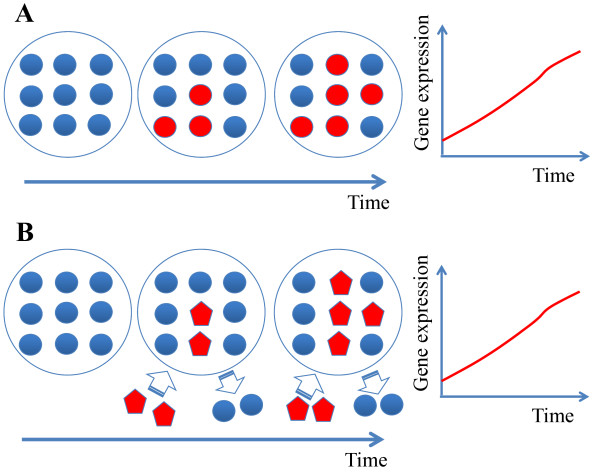
** Changes in cell demographics can result in gene expression.** Two scenarios which result in similar gene expression changes: (**A**) The cell type(s) within the sample are unchanged, but, over time, inactivated cells (colored blue) become activated and express a marker gene (colored red); (**B**) A second cell type already actively expressing the marker gene (red colored pentagons) migrates into the sample. The change in the marker gene expression is similar in both cases but results from a different reason.

Microarray studies quantify genome wide changes in gene expression and have a variety of applications - from tracing allele ancestry as species evolve [1] to the development of genome-based personalized medicine [2]. A major challenge in the microarray analysis of tissue collected *in vivo* is that often the perceived gene regulation is the result of changes in the populations of particular cell types as opposed to an actual change in transcriptional activity (see Figure 1). Particularly in situations which invoke the immune response, as the cell count of various lymphocytes change within the tissue, they bring with them their own unique quantities of RNA [3]. This leads to large changes in the copy number of RNA transcripts and can lead to the false perception of increased transcriptional activity. 

Several bioinformatics tools exist to identify the cause and effect of changes in gene regulation, with gene set enrichment analysis (GSEA) [4] and gene ontology (GO) enrichment [5] being the most popular, and there are several other web-based platforms with improvements or variations of these analytical tools [6-8]. GSEA relies on a database of reference gene lists which were previously determined to be regulated under several conditions (e.g., by transcription factors, chemical and genetic perturbations, or between healthy and diseased states). GSEA determines which reference list - if any - has statistically significant, concordant regulation. Although very useful for linking gene expression to specific transcription factors or identifying similarities between diseases, this tool does not include cell specific data at this time. The other popular alternative, GO, relies on a controlled vocabulary to describe the biological role of genes and their products. It is often accurate in predicting the local phenotype from gene expression data (e.g., inflammation annotations are highly enriched in samples from inflamed tissue [9]). However, cell specific GO annotations are often overwhelmed by more ubiquitous terms in the GO annotation hierarchy.

Additionally, some algorithms exist to unmix cellularly heterogeneous gene expression data into expression profiles for each cell type [10,11] but generally either the number of cell types must be known *a priori* or cell counts must be determined. The Gene Expression Barcode [12] and BioGPS [13] web platforms provide tissue specific gene expression data and allow researchers to compare gene expression between different tissues in their databases. However, these tools do not provide a means to relate user-generated sets of differentially expressed genes to specific cell types. Hence, to facilitate the proper interpretation of genomic regulation from *in vivo* microarray data, we developed CTen to determine if the observed gene regulation is the result of changes in the cellular make up of the sample.

Two principles guided the development of our highly expressed cell-specific (HECS) gene database and the CTen website's interface. First, basal gene expression levels strongly differ between cell types [3,14]. By analyzing gene expression across several cell types and tissues, we can select genes with very high expression in a limited number of cell types. In turn, each cell type has a collection of HECS genes to act as a cell-specific signature. Thus for any user generated list of genes, we can determine if the number of HECS genes for a particular cell type is greater than the number expected by chance.

The second principle, which led us to optimize CTen's interface for gene expression clustering studies, is the observation that changes in messenger RNA levels due to cell migration or variances in sample collection techniques result in conserved expression patterns in microarray data. Several clustering strategies, including hierarchical clustering and the weighted gene corregulation network algorithm (WGCNA) [15], have been developed to identify gene expression patterns which are conserved temporally or across experimental groups. By combining clustering with cell type enrichment, CTen can address a major challenge in biology today; namely separating gene expression from cellularly heterogeneous RNA samples into clusters representing differential transcriptional activity and clusters representing changes in gene expression due to cell migration.

Here, we first describe the construction of the HECS database and discuss the workflow behind the CTen website's design. We then validate CTen’s ability to correctly identify the appropriate cellular signature and evaluate the benefits of users requiring increasingly strict enrichment scores. We motivate the use of CTen using genes differentially expressed in the lungs of mice infected with influenza virus, and, lastly, provide an illustrative example promoting the use of CTen for detecting changes in the cellular demographics and the critical role this plays in functional enrichment and gene network inference studies.

## Implementation

### The HECS database construction

We downloaded from BioGPS [13] gene expression data from 96 mouse and 84 human tissues/cell types (Mouse MOE430 Gene Atlas and Human U133A/GNF1H Gene Atlas; a complete list of all cell types used is available in the Additional file 1). The expression values were averaged over the biological replicates (2 per cell type) and, for each cell type, a transcript was identified as a HECS gene if one of its corresponding probes had an expression value (averaged over the 2 replicates) at least 15x or 10x greater than the median expression value of the probe for all cell types in the mouse and human datasets, respectively. Next, probe identifiers were matched to their Entrez Gene IDs and official gene symbols using the Affymetrix Mouse Genome 430 2.0 Array (mouse4302 version 2.5.0) and Affymetrix Human Genome U133 Set (hgu133a version 2.5.0) annotation files available from Bioconductor [16]. The final step was to remove redundant Entrez Gene IDs assigned as HECS genes to the same cell type (due to multiple probes mapping to the same gene). The CTen database is available for download under the "Database Info" tab on the CTen website.

### Threshold selection

**Figure 2:**
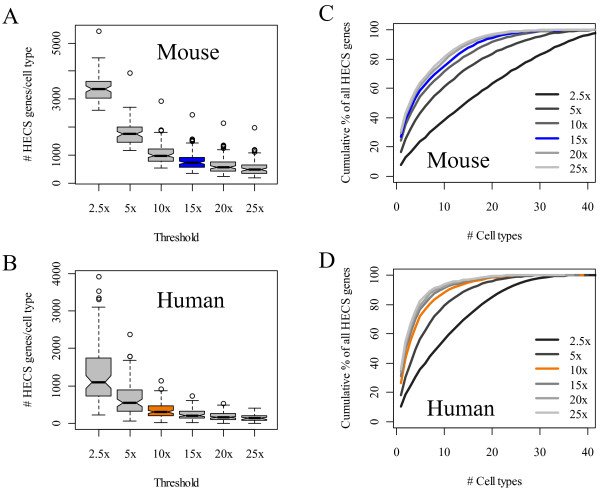
** The effect of threshold selection on the number and uniqueness of HECS genes.** The distributions of the number of HECS genes per cell type as the threshold criteria used to define a HECS gene is raised from 2x to 25x the median expression value across all cell types for the (**A**) mouse and (**B**) human gene expression data. To quantify uniqueness, we determined the percentage of HECS genes that were mapped to *n* or fewer cell types (i.e., the cumulative %) for the (**C**) mouse and (**D**) human gene expression data for different threshold values. The results corresponding to the threshold values selected in the current implementation of CTen are colored blue and orange for the mouse and human data, respectively.

Importantly, as stated above, preset cutoffs were used in developing the mouse and human HECS databases. These cutoffs (15x and 10x the median expression level for a probe across all cell types) were selected to balance the quantity of genes with the uniqueness of the genes assigned to each cell type. Uniqueness was quantified by determining the percentage of genes identified as a HECS gene for *n* or fewer cell types. As seen in Figure 2A-B, raising the cutoff caused a sharp reduction in the number of genes but significantly improved the uniqueness (Figure 2C-D) of the genes assigned as HECS genes to each cell type. Increasing the cutoff for the mouse data beyond 15x did not significantly improve uniqueness and only served to limit the number of HECS genes per cell type to act as cell signatures. For the cutoffs considered for the human data, a cutoff of 15x slightly improves the uniqueness but the number of HECS genes per cell type became prohibitively small. Thus, the HECS expression threshold requirement was reduced to 10x the median expression value in the human dataset to ensure that all cell types are represented.

At the cutoff values selected (emphasized in Figure 2A-B in blue (mouse) and orange (human)), even when applying a more stringent expression requirement, the number of HECS genes per cell type remains significantly higher in the mouse data (average number of HECS gene per cell = 794 in mouse and only 351 for human derived cell types). In terms of uniqueness of the HECS genes (emphasized in Figure 2C-D in blue (mouse) and orange (human)), we find that 55.8% of human HECS genes are exclusive to 3 or fewer cell types, while 53.3% of mouse HECS genes are limited to 4 or fewer cell types.

We emphasize that for both the mouse and human HECS databases, for values greater than 10x the median gene expression, the number of HECS genes per cell type and the identity of the HECS genes do not change significantly. Thus, cutoff selection within the ranges considered should not strongly bias any results from enrichment analysis. We validated this by showing that CTen's performance was independent of the precise threshold selected. We reconstructed the HECS databases for several different thresholds and then used the receiver operating characteristic (ROC) curve to determine if changing the threshold affected CTen's performance in terms of the true positive rate versus the false positive rate. As seen in Additional file 2 and Additional file 3, CTen's performance is robust to the precise threshold used for developing the HECS gene databases.

### The HECS genes are highly unique to each cell type

**Figure 3:**
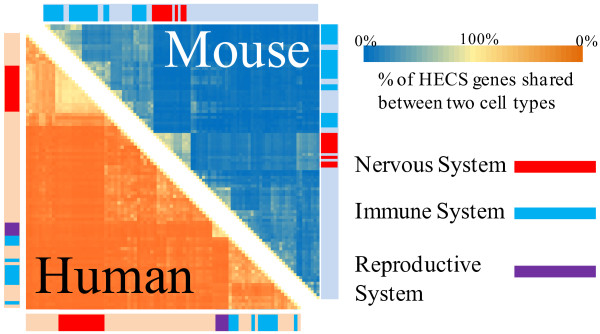
** HECS genes shared between different cell types.** A heatmap of the percentage of HECS genes shared between any two cell types in the mouse (upper triangle) and human (lower triangle) databases. Ticks adjacent to the heatmap denote cell types belonging to the nervous (red), immune (blue), or reproductive systems (purple).

We also determined the percentage of HECS genes shared by any two cell types within the human and mouse databases. As seen in Figure 3, the vast majority of cell types have highly distinct sets of HECS genes, with two mouse cell types sharing an average of only 16.1% HECS genes, while human cell types share an average of 11.6%. The two groups of cell types which share the most HECS genes in both mouse and human datasets belong to the nervous and reproductive systems (denoted by red and purple ticks beside the heatmap in Figure 3). Immune cells in different cell states also share the majority of their HECS genes (e.g., human CD8+ T-cells and CD4+ T-cells share 90.4% of their HECS genes) but the number of HECS genes shared between two different immune cells (e.g., B-cells versus T-cells) is generally less than 50% ( Additional file 4 provides a more detailed heatmap). In all, the strategy behind the development of the HECS database ensures that HECS genes are limited to a few cell types - characterizing a signature for each tissue. Therefore, the HECS database provides a powerful means of identifying cell/tissue specific enrichment in user gene lists.

### Data preprocessing and calculating the enrichment score

**Figure 4:**
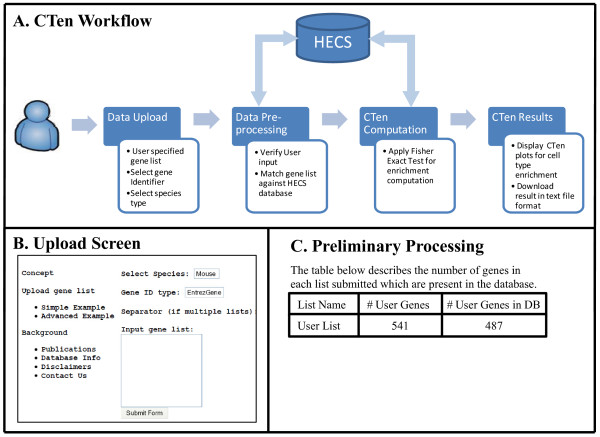
** Overview of a CTen session.** CTen has been designed with a user-friendly interface to allow for rapid analysis of several gene lists, simultaneously. Panel (**A**) illustrates the workflow between the user, CTen interface and the HECS database. (**B**) At the upload screen, users can copy and paste their gene lists straight from spreadsheet software (e.g., Excel) and select the appropriate parameters (species, gene identifier and the separator used if multiple lists are being uploaded). (**C**) The data goes through preliminary processing to ensure the gene list(s) is parsed properly and that the supplied genes are found in the HECS database. Once the user list passes preliminary processing, the enrichment of all cell types in the HECS database is calculated.

A minimal amount of preprocessing is applied to the user supplied gene list to ensure that, first, the list is properly parsed, and second, the user supplied genes are found in the HECS database. The workflow of the CTen website is shown in Figure 4A. At the upload screen (Figure 4B), users can upload a list of either gene symbols or Entrez gene IDs, and optionally upload multiple lists at once by choosing the appropriate format (the CTen webpage provides a single and multi-list example). The gene list is processed to determine the number of unique user genes found in the database and if the list does not appear to be one of the two gene identifiers stated above or the inappropriate format was selected, the website shows a parsing error screen and asks the user to ensure that the proper identifier is selected. If there are no parsing errors, CTen produces a table showing the number of unique user genes mapped in the CTen database for each uploaded list (Figure 4C). If no user genes are found in the database, CTen produces another error, "*No genes found in the database*" and the user is asked to reevaluate the uploaded gene list. Should CTen not detect either of these errors, the option to continue to enrichment appears and the user can complete their analysis.

Using the one-sided, Fisher's Exact test for enrichment, the enrichment score returned from CTen is the -log10 of the Benjamini-Hochberg (BH) adjusted P-values (all calculations are performed in R [17]). Although the enrichment score is a statistic in origin (indeed the enrichment scores could be used to control the false discovery rate), we advise users to consider the enrichment score to be a ranking and to not apply a strictly statistical understanding of the number. This is due to the sensitivity of the score to the size of the gene list being analyzed, and we show in detail in the Results and Discussion that ranking the results allows for easier interpretation. The appropriate contingency tables are constructed using the intersection of the user list and the HECS genes for each cell type. The gene universe (or gene background) against which the enrichment is calculated is currently defined to be all of the genes annotated in the human or mouse arrays defined above. Importantly, the enrichment scores for each gene list are calculated separately.

When only a single list is processed, a radar map of the enrichment scores is produced but in the case of multiple gene lists being supplied, P-values between each list cannot be compared since the length of the gene lists differ. So we developed a "weighted-ranking" strategy in which the enrichment scores for the 10 most enriched cell types in each list are scaled by the maximum enrichment score for that list. The enrichment scores of cell types either not present in the top 10 or present in the top 10 but with enrichment score of less than 2 are excluded. This procedure selects only the most enriched cell types for each list and allows us to visualize whether the enrichment scores of the top cell types were similar or if one cell type's enrichment score was dominant. The influenza-infected lung tissue example and the advanced use-case in the Results and Discussion illustrate CTen's output for single and multi-list analyses.

Finally, for both single- and multi-lists analyses, the final enrichment scores for all cell types can be downloaded for further processing by the users.

## Results and discussion

### CTen correctly identifies cell types

To assess CTen’s ability to identify the correct cell type associated with gene expression data, we used an independent database of cell-specific gene expression (GNF1M_plus_macrophage_small dataset from BioGPS; abbreviated GNFM1) to develop several lists of genes which were highly expressed in select cell types. This data set is an interesting test case for CTen because the differences in the experimental protocol tests CTen's performance when using different microarray technologies and biological conditions. In the GNF1M experiment, they used mice which were ~2 weeks older (compared to the mice used to develop the Mouse MOE430 Gene Atlas data set), used a different ratio of male and female mice, and employed custom microarray slides (GPL1037) [3]. For several cell types (5 tissues and 3 lymphocytes; 2 lymphocytes in different cellular states), we selected the top 5% of the most highly expressed probes. Entrez Gene IDs were mapped using the annotation files available from BioGPS, and the resulting lists analyzed in CTen.

**Figure 5:**
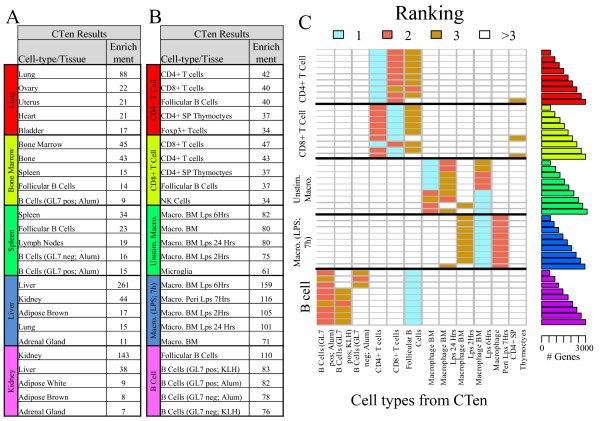
** CTen validation and robustness of the enrichment score rankings.** From an additional cell-specific gene expression database, we took the top 5% most highly expressed genes for five kinds of tissue (lung, bone marrow, kidney, spleen and liver) and three lymphocytes in different cellular states (CD4^+^ and CD8^+^ T cells, B cells and macrophages that were unstimulated or collected 7 hours after simulation with LPS). Each list was analyzed in CTen and we show the top 5 enrichments scores for (**A**) the tissue (**B**) lymphocyte test lists. To evaluate the robustness of these results, we repeated this procedure for the top 2-10% most highly expressed genes in each cell type. The enrichment scores from CTen were ranked from highest to lowest, and (**C**) the heatmap illustrates the top 3 most enriched cell types identified by CTen (columns) for each lymphocyte data tested (row labels). The bar plot on the right hand side summarizes the number of genes per test list. The heatmap for the tissue data is available as Additional file 5.

We found that CTen consistently ranked the correct cell type the highest for each tissue tested (Figure 5A) and, with the exception of bone marrow, there was a large difference in the scores between the first and second most enriched cell types. Not surprisingly, bone marrow was identified as being highly enriched for bone. For the lymphocyte gene lists (Figure 5B), CTen not only identified the correct lymphocyte but most often identified the correct cellular state of the lymphocyte as being the top ranked cell type. Only for the unstimulated macrophages did CTen rank the inappropriate cellular states the highest. Thus, from independent, cell-specific gene expression data, we confirmed that CTen provides clear guidance in relating gene expression data to the appropriate cell type.

### Ranking of the enrichment scores are robust

As with any analysis, small changes in experiment parameters should not greatly change the outcome. P-values from the Fisher Exact test are very sensitive to changes in the size of the gene list, but for many enrichment analyses, it has been observed that the rankings of the enrichment scores are very robust [7,18]. Here, we asked if CTen could robustly rank the correct cell types by repeating the procedure described above - now using a list of the top 2, 3,…, 10% most highly expressed genes for the selected tissues and lymphocytes, resulting in 90 test lists. The different sizes of the lists simulate different differential expression criteria during gene expression analysis. As shown in Figure 5C, although the sizes of the gene lists (and the underlying enrichment scores) vary considerably, CTen most often ranks the appropriate cell type the most highly. CTen was also able to identify the proper cell state of the lymphocytes as well although unstimulated macrophage data was assigned to *bone marrow macrophages collected 6 h after exposure to lipopolysaccharide (LPS)* 4 out of 9 times. CTen performed even better for the tissue data, always ranking the appropriate tissue the highest ( Additional file 5). In all, CTen can accurately identify a broad range of cell types and very often identify the cellular state as well. The results are very robust to changes in the length of the test data, which can be equated to changes in the cutoff criteria used during microarray analysis.

### Minimizing the false positive rate

**Figure 6:**
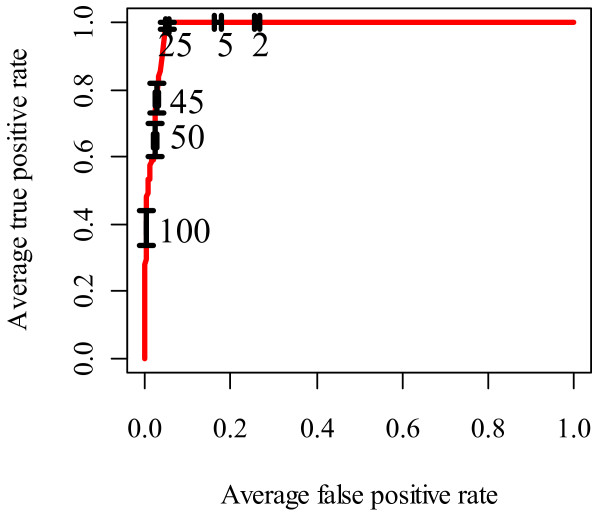
** CTen's performance for different levels of enrichment.** Using the same test lists behind the results shown in Figure 5C, we constructed an ROC curve to evaluate CTen's classification performance for different levels of the enrichment score. The error bars depict the 95% confidence interval of the ROC curve for the enrichment scores shown.

While CTen accurately identified the appropriate cell type as having the highest enrichment score, we think it's important to provide a comprehensive analysis of CTen's accuracy for select cutoff values of the enrichment score. Using the same test lists developed above for Figure 5C, we used the receiver operating characteristic (ROC) curve to identify what level of enrichment was necessary to maximize the sensitivity (true positive rate, TPR) while minimizing the false positive rate (FPR) (Figure 6). Demanding a minimal enrichment score of 2 provides a low FPR and, indeed, we found that for randomly generated lists of genes, CTen rarely assigned scores above 2 ( Additional file 6). But we see here, raising the enrichment score cutoff from 2 to 25 greatly minimizes the FPR without sacrificing the TPR. Requiring enrichment scores above 25 only reduces the sensitivity of the analysis. A similar analysis to this was performed using the two databases from which CTen was constructed resulting in nearly identical ROC curves ( Additional file 2 and Additional file 3). These curves also suggest enrichment scores of 20–25 to optimally minimize the FPR for mouse data, but slightly lower enrichment scores (15 to 20) offer optimal performance for human data. It should be noted that these performance results are dependent on the size of the gene list. Thus, for gene lists which are hundreds to thousands of genes in number, a minimum enrichment score of 2 is recommended, but scores of 20–25 appear to offer optimal performance.

### CTen versus GO analysis of influenza infected lung tissue

**Figure 7:**
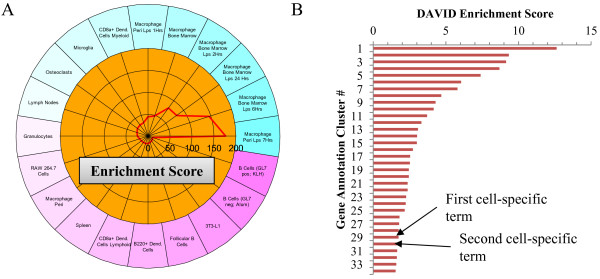
** CTen versus GO analysis.** A list of upregulated genes in lung tissue collected from mice infected with the influenza virus is analyzed in (**A**) CTen and (**B**) DAVID. The first cluster to have a cell specific term is ranked 29th in the DAVID analysis. A complete list of the terms belonging to each cluster is available in Additional file 7.

Using a list of genes found to be upregulated in lung tissue collected from mice infected with influenza virus (microarray data unpublished; the gene list is available on the CTen website under the "Simple Example" tab), we compared the results of a CTen analysis to a GO analysis using DAVID [7]. Using the CTen website, we find a very high enrichment of bone marrow derived and peritoneal macrophages (Figure 7A), both of which have been exposed to lipopolysaccharide (LPS) and collected at different time points. Macrophage migration to the site of infection is one of the first steps in coordinating the innate immune response [19]. Both LPS exposure [20] and influenza infection [21] induces the activation of the Toll-like receptor pathways, and macrophages are often susceptible to influenza infection themselves [22]. Thus, an increase in macrophage numbers is consistent with previously published studies [23] and the observation of the resulting cell type as "*macrophage exposed to LPS*", indicates that the macrophages have possibly become infected with the influenza virus as well. 

DAVID uses modules of related biological terms to interpret large gene lists into a meaningful biological context and reports the scores of each module as the -log10 of the average P-value for each term within the module [24]. Using the default settings, DAVID identifies the Toll-like receptor pathway (Figure 7B, Cluster #1) as the most significant cluster of annotations (Enrichment score: 12.62; full results available in Additional file 7). However, the clusters indicating enhanced macrophage presence have a low significance (Cluster #29; enrichment score: 1.74) and are very closely followed by a T-cell related cluster (Enrichment score: 1.68). Taken together, these results indicate that although both analyses can identify aspects of the cellular state of the sample, CTen is better suited to identify the known changes in the cellular demographics of the RNA samples.

### Advanced use-case: distinguishing changes in lymphocyte cell count from gene transcription

The most exciting potential of CTen is that, when applied to clustering studies, cell type enrichment analysis can be used to approximate the evolution of local cellular demographics. Our laboratory's research is primarily focused on reconstructing the host response during an influenza infection [25]; a goal which requires us to be able to integrate local intracellular signaling (Toll-like receptor/RIG-I/NFκB pathways) with the coordinated migration, infiltration, and activity of macrophages, T-cells, B-cells, and other immune related cell-types. Being able to resolve the various cell types present in a sample from microarray data would greatly facilitate discovery in a broad range of *in vivo* studies.

**Figure 8:**
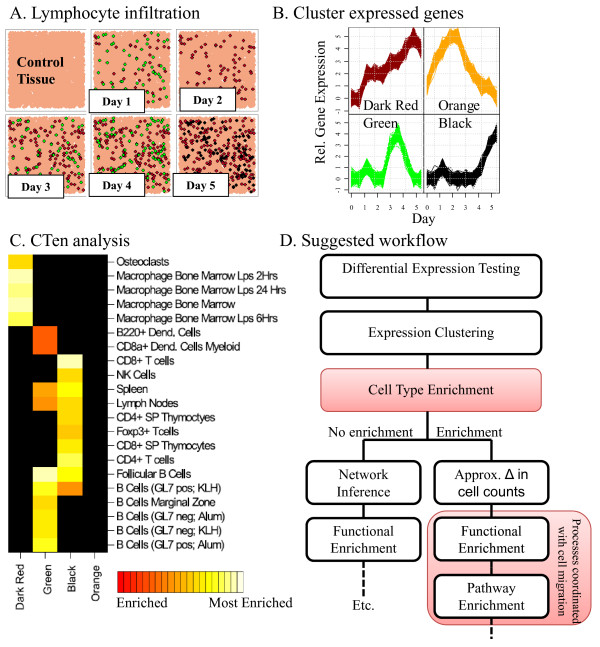
** Detecting changes in lymphocyte cell counts and the appropriateness of follow-up analyses using CTen.** (**A**) In infected tissue**,** as B cells (green), T cells (black), and macrophages (dark red) migrate into the sample area, they bring with them their own unique quantities of RNA, resulting in conserved expression patterns proportional to the cell type's increased (or decreased) presence. Here, we illustrate the potential results from a clustering study using WGCNA [15], resulting in (**B**) 4 clusters with unique temporal expression profiles; 3 of which are highly correlated to the changes in the number of lymphocytes shown in (A). (**C**) Analyzing the genes present in each cluster, CTen can distinguish which clusters represent gene expression and which represent cell migration, and determine the cell type responsible for the observed gene regulation. Using a "weighted-ranking" strategy (see text), CTen produces a heatmap showing the most enriched cell types for each cluster. Based on the CTen result, only the orange cluster is appropriate for further analysis using traditional bioinformatic techniques while the green, black, and dark red clusters reflect the relative changes in the number of lymphocytes during the infection. (**D**) We propose a new analytical workflow strategy which ensures that continued analysis of *in vivo* microarray data properly identifies events which may be coordinated with (or coordinating) cell migration.

Figure 8 illustrates the proposed strategy for identifying cellular signatures in *in vivo* data and its implications for *in vivo* microarray based studies. In this illustration, microarray data was assembled over a span of 5 days from the lungs of mice infected with influenza virus on day 0 (lung tissues are illustrated in Figure 8A). After normalizing and differential expression testing, four gene clusters (Figure 8B) were identified using the user's preferred clustering tool.

In this case, we are illustrating potential results from using the WGCNA package [15], which applies color labels to each cluster. The genes for each cluster can be uploaded and analyzed in one session to identify the most enriched cell types in each cluster. In Figure 8C, we find that macrophages are highly enriched in the dark red cluster while several categories of B- and T-cells (CD8+ T-cells) are the most enriched cell types in the green and black clusters, respectively. Interestingly, the orange cluster is not enriched for any cell type, and we would conclude that transcripts in the orange cluster represent differential gene expression due to transcriptional differences between the samples (as opposed to difference in the cellular makeup of the samples) and are suitable for further analysis using traditional approaches. The dark red, green and black clusters can be further analyzed for pathway or functional enrichment to identify processes that may be coordinated with cell migration. This result may also help researchers decide the appropriateness of additional analyses. Some analyses, such as gene network inference, will have to carefully consider how to remove the effects of cell migration prior to network construction. Furthermore, the green, black and dark red clusters' gene expression is highly correlated to the corresponding lymphocyte's cell count change. Thus, we may be able to infer the relative changes in the B cell, T cell and macrophage count in the infected tissue.

In all, this example illustrates how CTen has been designed to facilitate the understanding of clustering results by identifying conserved expression patterns that are the result of changes in the numbers of a particular cell type, providing critical guidance for selecting additional analyses for each gene set and allowing users to infer changes in cellular demographics between samples. Based on the CTen enrichment platform, we propose a novel analytical workflow for *in vivo* microarray, as illustrated schematically in Figure 8D, which ensures that enriched biological pathways and processes identified in a set of differentially expressed genes can be interpreted in the proper cellular context.

## Conclusions

In conclusion, CTen can effectively distinguish between active gene transcription and apparent gene expression resulting from differences in the numbers of select cell types in microarray data. Furthermore, we provide a novel research workflow which helps to ensure that gene expression is interpreted in the proper biological context. We will continuously improve the enrichment algorithm so that a larger number of gene lists can be processed simultaneously (currently, users are limited to 20 gene lists in a single session). Recently, a gene set enrichment analysis based on the degree of pairwise correlation within a given gene set was shown to successfully relate samples to their corresponding tissue [26]. No simple interface is available yet for researchers, but it will be interesting to compare the performance between these two approaches in the near future. Additionally, we plan to introduce additional cell specific gene expression datasets so users can compare the results from different databases. And finally, while the examples focused on lymphocyte migration, CTen can be used in several other scenarios; for example, comparing excised tissue to ensure homogeneity between tissue samples.

## Availability and requirements

Project name: CTen

Project home page: http://www.influenza-x.org/~jshoemaker/cten/

Operating system: Platform independent

Programming Language: PHP and R

Other requirements: None

License: EULA

## Competing interests

No competing interests to declare.

## Authors' contributions

JES designed the project, built the database and wrote the manuscript. TJL, SG, YK and HK revised the manuscript. JES, TJL, SG, YM, and HK implemented the website and YM maintains the public server. All authors have read and approved the final manuscript.

## Supplementary Material

Additional file 1 A list of the cell types currently available in CTen. Click here for file

Additional file 2 ** The enrichment performance of the mouse HECS database for select HECS criteria and enrichment scores.** We evaluated (1) does the precise cutoff for defining a HECS gene affect the enrichment performance and (2) for each cutoff, what values of the enrichment score seems to best minimize the false positive rate (FPR) without impacting the true positive rate (TPR). We reconstructed the HECS database by defining the HECS assignment threshold as (A) 5, (B) 10, (C) 15, and (D) 20 times the median. Then, from the Mouse MOE430 Gene Atlas dataset, we took the top 10% of the most highly expressed genes for each cell type. From this 10%, we randomly sampled between 500 to 4000 genes 3 times to create 288 gene lists. Using the same procedures described in the CTen implementation, these lists were analyzed for cell type enrichment for each HECS database constructed. The ROC curve illustrates the that sensitivity (TPR) and the FPR are not greatly affected by the HECS assignment threshold selected. Furthermore, on each figure, we show the performance expected for selected values of the enrichment score. We see that selecting enrichment scores of 2 or higher results in a reasonably low FPR but this can be significantly improved by demanding enrichments scores of ~25 before the TPR is affected. Click here for file

Additional file 3 ** The enrichment performance of the human HECS database for select HECS criteria and enrichment scores.** We evaluated (1) does the precise cutoff for defining a HECS gene affect the enrichment performance and (2) for each cutoff, what values of the enrichment score seems to best minimize the false positive rate (FPR) without impacting the true positive rate (TPR). We reconstructed the HECS database by defining the HECS assignment threshold as (A) 5, (B) 10, and (C) 15 times the median. Then, from the Human U133A/GNF1H Gene Atlas dataset, we took the top 10% of the most highly expressed genes for each cell type. From this 10%, we randomly sampled between 500 to 4000 genes 3 times to create 252 gene lists. Using the same procedures described in the CTen implementation, these lists were analyzed for cell type enrichment for each HECS database constructed. The ROC curve illustrates the that sensitivity (TPR) and the FPR are not greatly affected by the HECS assignment threshold selected. Furthermore, on each figure, we show the performance expected for selected values of the enrichment score. We see that selecting enrichment scores of 2 or higher results in a reasonably low FPR but this can be significantly improved by demanding enrichments scores of ~20 before the TPR is affected. Click here for file

Additional file 4 A heatmap of the percentage of HECS genes shared by any two cell types in the mouse (upper right) and human (lower left) databases. Click here for file

Additional file 5 ** The highest ranked cell types identified by CTen.**Using the GNF1M_plus_macrophage_small dataset from BioGPS, the top 2-10% most highly expressed genes for tissues shown were analyzed in CTen. The enrichment scores from CTen were ranked from highest to lowest, and the heatmap illustrates the top 3 most enriched cell types (columns) for each lymphocyte data tested (row labels). BM = bone marrow. Click here for file

Additional file 6 ** Expected enrichment scores for random gene lists.** We analyzed in CTen 150 lists of 100–400 randomly selected IDs for (A) mouse and (B) human Entrez Gene IDs - this resulted in a distribution of enrichment scores. The distributions were fit to a gamma distribution using the MASS package in R. Here, we show the density histogram and fitted gamma function (left hand axis) and the probability distribution function (right hand axis). The red bar highlights the enrichment score which is 95% confidently above 0 (α = 0.95 at enrichment scores of 1.66 and 1.67 in the mouse and human data, respectively). Click here for file

Additional file 7 A list of genes upregulated in mouse lungs which have been infected with influenza virus and the full results of analyzing this list in DAVID. Click here for file

## References

[B1] Hacia JG Fan JB Ryder O Jin L Edgemon K Ghandour G Mayer RA Sun B Hsie L Robbins CM Determination of ancestral alleles for human single-nucleotide polymorphisms using high-density oligonucleotide arrays Nat Genet 1999 22 2 164 167 10.1038/9674 10369258

[B2] Shao L Fan X Cheng N Wu L Xiong H Fang H Ding D Shi L Cheng Y Tong W Shifting from population-wide to personalized cancer prognosis with microarrays PLoS One 2012 7 1 e29534 10.1371/journal.pone.0029534 22295060 PMC3266237

[B3] Su AI Wiltshire T Batalov S Lapp H Ching KA Block D Zhang J Soden R Hayakawa M Kreiman G A gene atlas of the mouse and human protein-encoding transcriptomes Proc Natl Acad Sci USA 2004 101 16 6062 6067 10.1073/pnas.0400782101 15075390 PMC395923

[B4] Subramanian A Tamayo P Mootha VK Mukherjee S Ebert BL Gillette MA Paulovich A Pomeroy SL Golub TR Lander ES Gene set enrichment analysis: a knowledge-based approach for interpreting genome-wide expression profiles Proc Natl Acad Sci USA 2005 102 43 15545 15550 10.1073/pnas.0506580102 16199517 PMC1239896

[B5] Ashburner M Ball CA Blake JA Botstein D Butler H Cherry JM Davis AP Dolinski K Dwight SS Eppig JT Gene ontology: tool for the unification of biology The Gene Ontology Consortium. Nat Genet 2000 25 1 25 29 10.1038/75556 PMC3037419 10802651

[B6] Chang JT Nevins JR GATHER: a systems approach to interpreting genomic signatures Bioinformatics 2006 22 23 2926 2933 10.1093/bioinformatics/btl483 17000751

[B7] da Huang W Sherman BT Lempicki RA Systematic and integrative analysis of large gene lists using DAVID bioinformatics resources Nat Protoc 2009 4 1 44 57 19131956 10.1038/nprot.2008.211

[B8] Kaimal V Bardes EE Tabar SC Jegga AG Aronow BJ ToppCluster: a multiple gene list feature analyzer for comparative enrichment clustering and network-based dissection of biological systems Nucleic Acids Res 2010 38 W96 102 10.1093/nar/gkq418 20484371 PMC2896202

[B9] Baas T Baskin CR Diamond DL Garcia-Sastre A Bielefeldt-Ohmann H Tumpey TM Thomas MJ Carter VS Teal TH Van Hoeven N Integrated molecular signature of disease: analysis of influenza virus-infected macaques through functional genomics and proteomics J Virol 2006 80 21 10813 10828 10.1128/JVI.00851-06 16928763 PMC1641753

[B10] Schwartz R Shackney SE Applying unmixing to gene expression data for tumor phylogeny inference BMC Bioinforma 2010 11 42 10.1186/1471-2105-11-42 PMC2823708 20089185

[B11] Shen-Orr SS Tibshirani R Khatri P Bodian DL Staedtler F Perry NM Hastie T Sarwal MM Davis MM Butte AJ Cell type-specific gene expression differences in complex tissues Nat Methods 2010 7 4 287 289 10.1038/nmeth.1439 20208531 PMC3699332

[B12] McCall MN Uppal K Jaffee HA Zilliox MJ Irizarry RA The Gene Expression Barcode: leveraging public data repositories to begin cataloging the human and murine transcriptomes Nucleic Acids Res 2011 39 D1011 1015 Database issue 10.1093/nar/gkq1259 21177656 PMC3013751

[B13] Wu C Orozco C Boyer J Leglise M Goodale J Batalov S Hodge CL Haase J Janes J Huss JW 3rd BioGPS: an extensible and customizable portal for querying and organizing gene annotation resources Genome Biol 2009 10 11 R130 10.1186/gb-2009-10-11-r130 19919682 PMC3091323

[B14] Eisenberg E Levanon EY Human housekeeping genes are compact Trends Genet 2003 19 7 362 365 10.1016/S0168-9525(03)00140-9 12850439

[B15] Langfelder P Horvath S WGCNA: an R package for weighted correlation network analysis BMC Bioinforma 2008 9 559 10.1186/1471-2105-9-559 PMC2631488 19114008

[B16] Gentleman RC Carey VJ Bates DM Bolstad B Dettling M Dudoit S Ellis B Gautier L Ge Y Gentry J Bioconductor: open software development for computational biology and bioinformatics Genome Biol 2004 5 10 R80 10.1186/gb-2004-5-10-r80 15461798 PMC545600

[B17] Team RDC R: A Language and Environment for Statistical Computing 2010 R Foundation for Statistical Computing, Vienna, Austria

[B18] da Huang W Sherman BT Lempicki RA Bioinformatics enrichment tools: paths toward the comprehensive functional analysis of large gene lists Nucleic Acids Res 2009 37 1 1 13 10.1093/nar/gkn923 19033363 PMC2615629

[B19] Medzhitov R Recognition of microorganisms and activation of the immune response Nature 2007 449 7164 819 826 10.1038/nature06246 17943118

[B20] Aderem A Ulevitch RJ Toll-like receptors in the induction of the innate immune response Nature 2000 406 6797 782 787 10.1038/35021228 10963608

[B21] Sun L Liu S Chen ZJ SnapShot: pathways of antiviral innate immunity Cell 2010 140 3 436 436 e432 10.1016/j.cell.2010.01.041 20144765 PMC3586550

[B22] Yu WC Chan RW Wang J Travanty EA Nicholls JM Peiris JS Mason RJ Chan MC Viral replication and innate host responses in primary human alveolar epithelial cells and alveolar macrophages infected with influenza H5N1 and H1N1 viruses J Virol 2011 85 14 6844 6855 10.1128/JVI.02200-10 21543489 PMC3126566

[B23] Reading PC Whitney PG Pickett DL Tate MD Brooks AG Influenza viruses differ in ability to infect macrophages and to induce a local inflammatory response following intraperitoneal injection of mice Immunol Cell Biol 2010 88 6 641 650 10.1038/icb.2010.11 20142836

[B24] da Huang W Sherman BT Tan Q Collins JR Alvord WG Roayaei J Stephens R Baseler MW Lane HC Lempicki RA The DAVID Gene Functional Classification Tool: a novel biological module-centric algorithm to functionally analyze large gene lists Genome Biol 2007 8 9 R183 10.1186/gb-2007-8-9-r183 17784955 PMC2375021

[B25] Fukuyama S Kawaoka Y The pathogenesis of influenza virus infections: the contributions of virus and host factors Curr Opin Immunol 2011 23 4 481 486 10.1016/j.coi.2011.07.016 21840185 PMC3163725

[B26] Chang JT Deriving transcriptional programs and functional processes from gene expression databases Bioinformatics 2012 28 8 1122 1129 10.1093/bioinformatics/bts112 22408194 PMC3324522

