# Supplementary figures and images for: CTen: a web-based platform for identifying enriched cell types from heterogeneous microarray data
Source: BMC Genomics. 2012 Sep 6;13:460. doi: 10.1186/1471-2164-13-460 (PMC3473317; doi:10.1186/1471-2164-13-460)

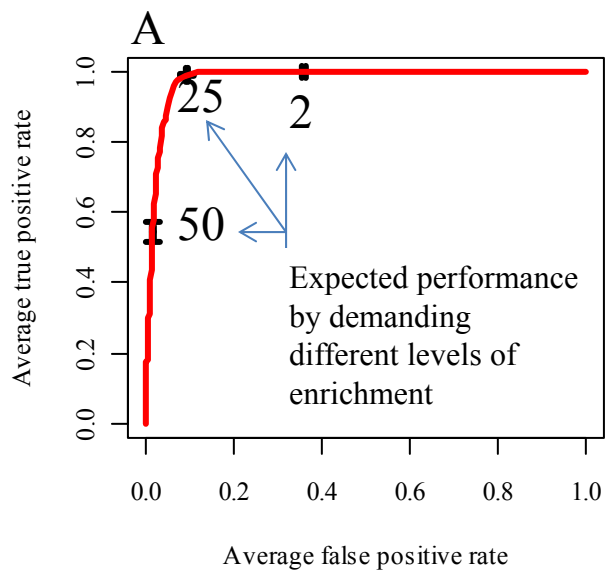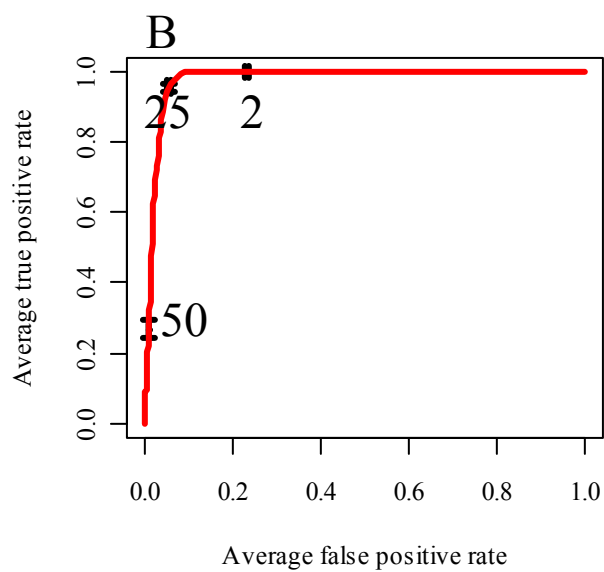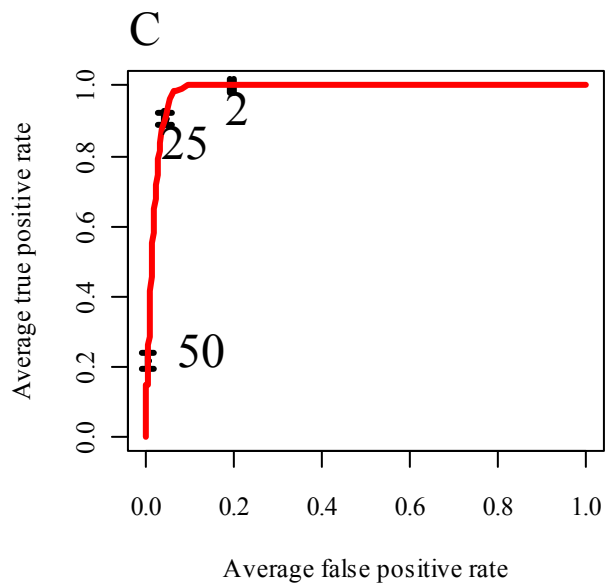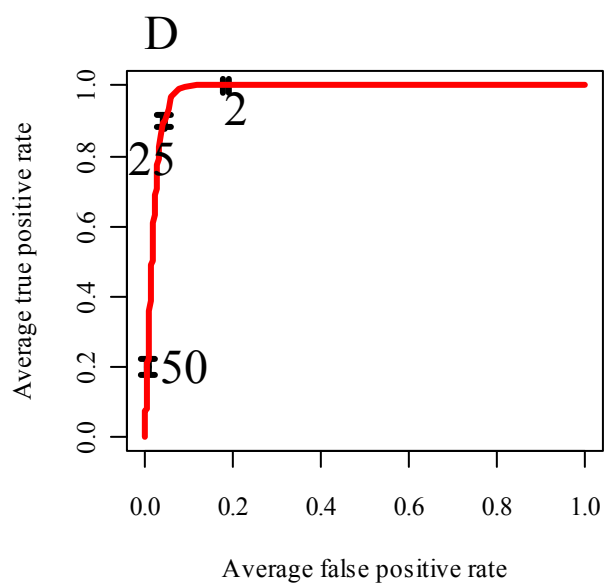

Supplement: Additional file 2 — The enrichment performance of the mouse HECS database for select HECS criteria and enrichment scores. We evaluated (1) does the precise cutoff for defining a HECS gene affect the enrichment performance and (2) for each cutoff, what values of the enrichment score seems to best minimize the false positive rate (FPR) without impacting the true positive rate (TPR). We reconstructed the HECS database by defining the HECS assignment threshold as (A) 5, (B) 10, (C) 15, and (D) 20 times the median. Then, from the Mouse MOE430 Gene Atlas dataset, we took the top 10% of the most highly expressed genes for each cell type. From this 10%, we randomly sampled between 500 to 4000 genes 3 times to create 288 gene lists. Using the same procedures described in the CTen implementation, these lists were analyzed for cell type enrichment for each HECS database constructed. The ROC curve illustrates the that sensitivity (TPR) and the FPR are not greatly affected by the HECS assignment threshold selected. Furthermore, on each figure, we show the performance expected for selected values of the enrichment score. We see that selecting enrichment scores of 2 or higher results in a reasonably low FPR but this can be significantly improved by demanding enrichments scores of ~25 before the TPR is affected. [file 1471-2164-13-460-S2.pdf]

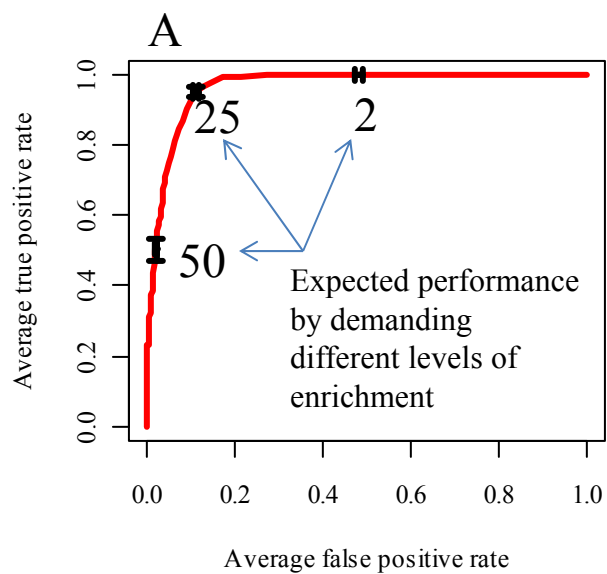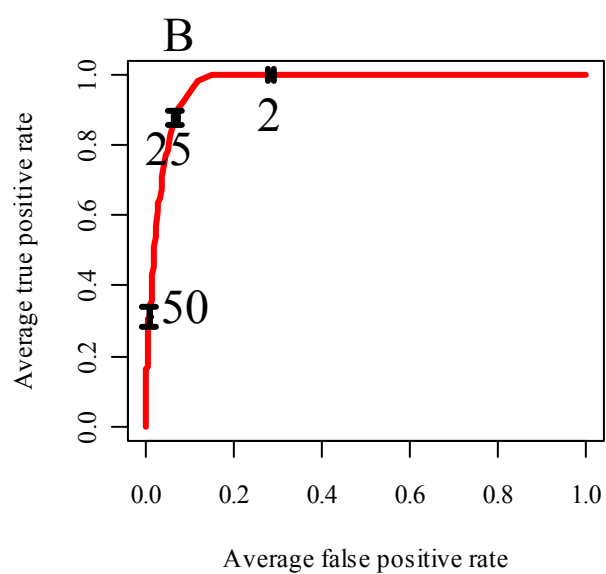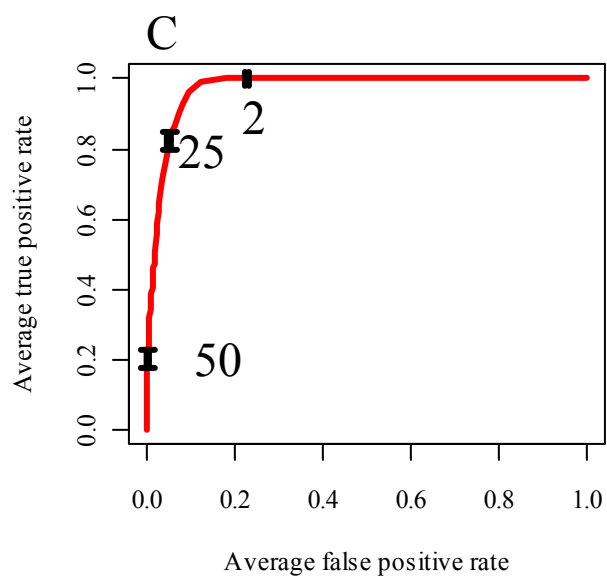

Supplement: Additional file 3 — The enrichment performance of the human HECS database for select HECS criteria and enrichment scores. We evaluated (1) does the precise cutoff for defining a HECS gene affect the enrichment performance and (2) for each cutoff, what values of the enrichment score seems to best minimize the false positive rate (FPR) without impacting the true positive rate (TPR). We reconstructed the HECS database by defining the HECS assignment threshold as (A) 5, (B) 10, and (C) 15 times the median. Then, from the Human U133A/GNF1H Gene Atlas dataset, we took the top 10% of the most highly expressed genes for each cell type. From this 10%, we randomly sampled between 500 to 4000 genes 3 times to create 252 gene lists. Using the same procedures described in the CTen implementation, these lists were analyzed for cell type enrichment for each HECS database constructed. The ROC curve illustrates the that sensitivity (TPR) and the FPR are not greatly affected by the HECS assignment threshold selected. Furthermore, on each figure, we show the performance expected for selected values of the enrichment score. We see that selecting enrichment scores of 2 or higher results in a reasonably low FPR but this can be significantly improved by demanding enrichments scores of ~20 before the TPR is affected. [file 1471-2164-13-460-S3.pdf]

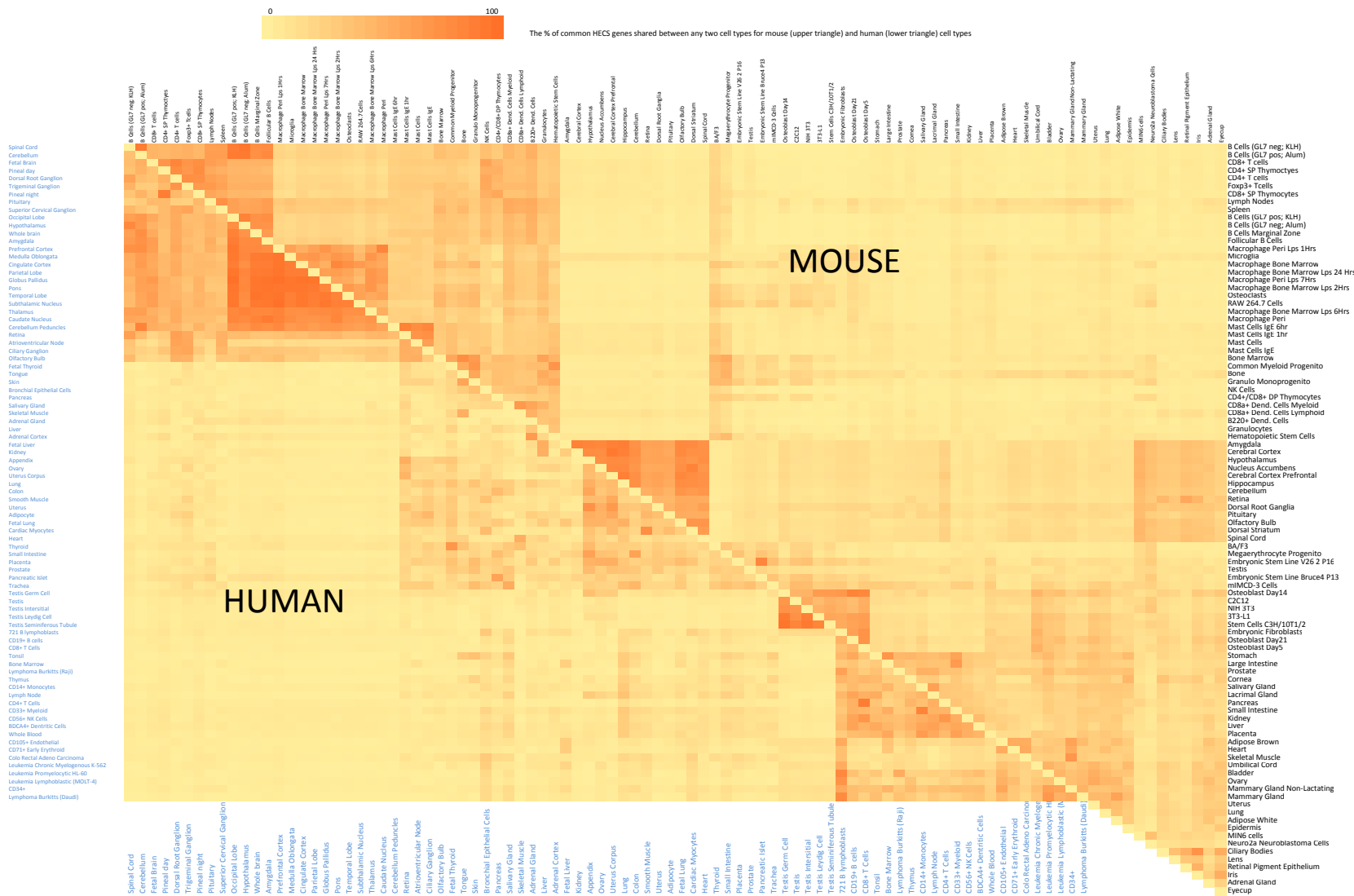

Supplement: Additional file 4 — A heatmap of the percentage of HECS genes shared by any two cell types in the mouse (upper right) and human (lower left) databases. [file 1471-2164-13-460-S4.pdf]

# Ranking

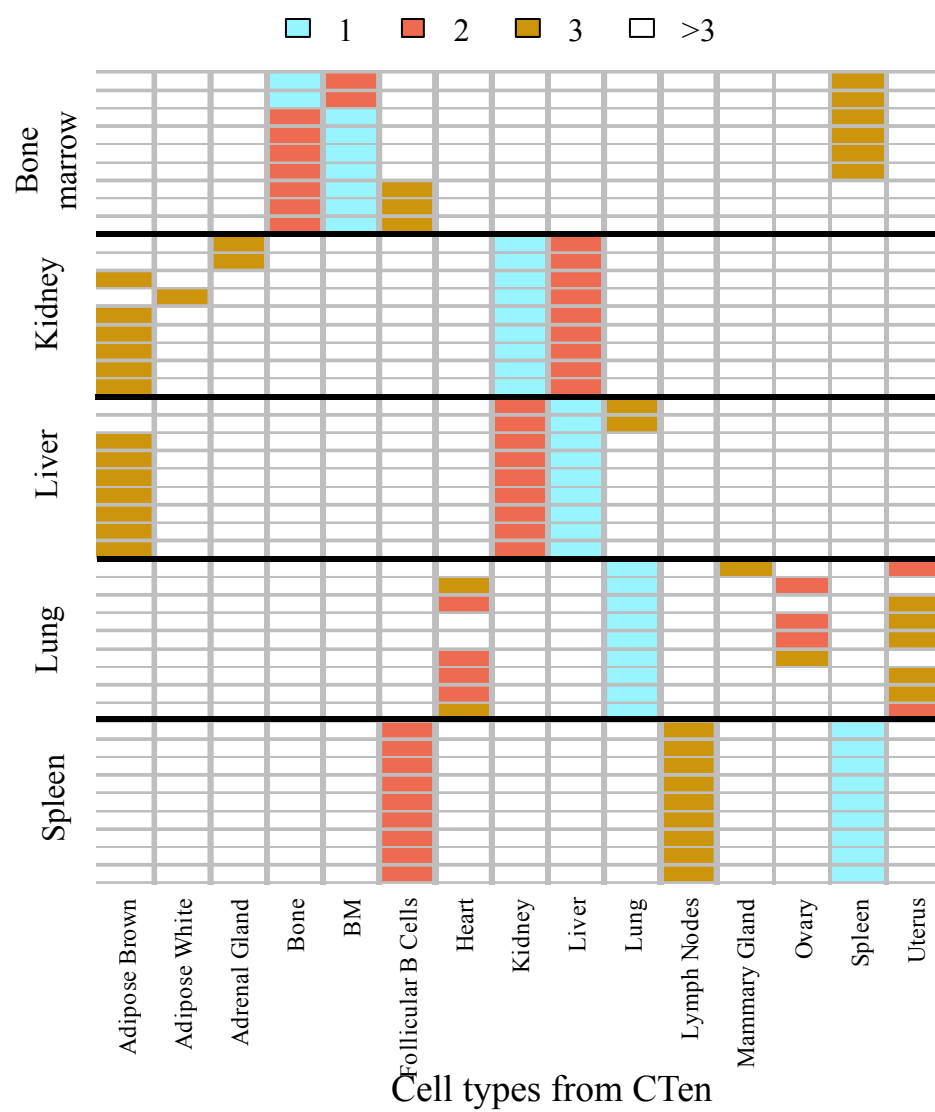

Supplement: Additional file 5 — The highest ranked cell types identified by CTen.Using the GNF1M_plus_macrophage_small dataset from BioGPS, the top 2-10% most highly expressed genes for tissues shown were analyzed in CTen. The enrichment scores from CTen were ranked from highest to lowest, and the heatmap illustrates the top 3 most enriched cell types (columns) for each lymphocyte data tested (row labels). BM = bone marrow. [file 1471-2164-13-460-S5.pdf]

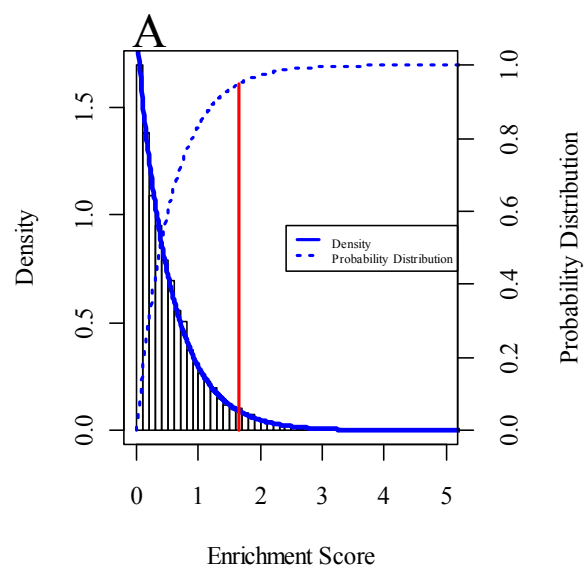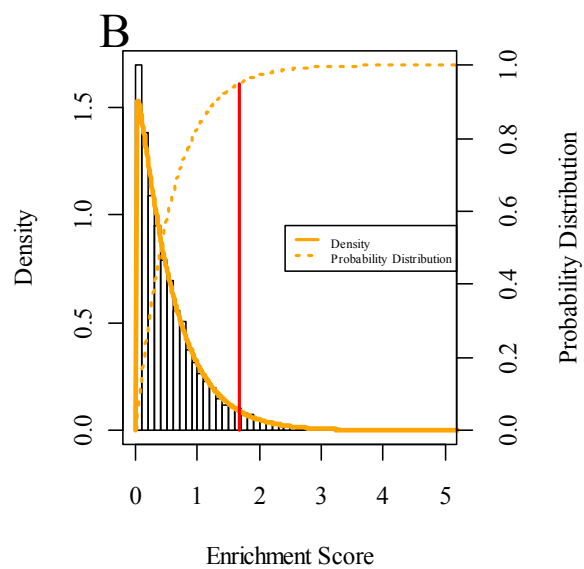

Supplement: Additional file 6 — Expected enrichment scores for random gene lists. We analyzed in CTen 150 lists of 100–400 randomly selected IDs for (A) mouse and (B) human Entrez Gene IDs - this resulted in a distribution of enrichment scores. The distributions were fit to a gamma distribution using the MASS package in R. Here, we show the density histogram and fitted gamma function (left hand axis) and the probability distribution function (right hand axis). The red bar highlights the enrichment score which is 95% confidently above 0 (α = 0.95 at enrichment scores of 1.66 and 1.67 in the mouse and human data, respectively). [file 1471-2164-13-460-S6.pdf]
